# A Character Segmentation Proposal for High-Speed Visual Monitoring of Expiration Codes on Beverage Cans

**DOI:** 10.3390/s16040527

**Published:** 2016-04-13

**Authors:** José C. Rodríguez-Rodríguez, Alexis Quesada-Arencibia, Roberto Jr. Moreno-Díaz, Carmelo R. García

**Affiliations:** Institute for Cybernetics, Campus de Tafira, Las Palmas de Gran Canaria, University of Las Palmas de Gran Canaria, 35017 Las Palmas, Spain; jcarlos@ciber.ulpgc.es (J.C.R.-R.); rmorenoj@dis.ulpgc.es (R.J.M.-D.); rgarcia@dis.ulpgc.es (C.R.G.)

**Keywords:** image processing, OCR (Optical Character Recognition), pattern recognition, industrial inspection, very high-speed computing, character segmentation, tracking

## Abstract

Expiration date labels are ubiquitous in the food industry. With the passage of time, almost any food becomes unhealthy, even when well preserved. The expiration date is estimated based on the type and manufacture/packaging time of that particular food unit. This date is then printed on the container so it is available to the end user at the time of consumption. MONICOD (MONItoring of CODes); an industrial validator of expiration codes; allows the expiration code printed on a drink can to be read. This verification occurs immediately after printing. MONICOD faces difficulties due to the high printing rate (35 cans per second) and problematic lighting caused by the metallic surface on which the code is printed. This article describes a solution that allows MONICOD to extract shapes and presents quantitative results for the speed and quality.

## 1. Introduction

The law states that any perishable products intended for human consumption must display the date after which their consumption is no longer considered safe. Canned beverages are no exception. This information is typically encoded on the base of the container or can as a set of characters. Interpreting this expiration code must be simple, effective, and free of ambiguities. Character visibility and legibility must also be ensured.

Comprehensive human verification of these printed labels is infeasible due to the overwhelming and monotonous nature of production and high labelling rate. The last few decades have seen significant advances for delegating such monitoring to machines, e.g., high-performance computing or development (and cost-lowering) of high-speed cameras capable of acquiring images at a high frequency. However, despite the numerous publications in the field of character recognition (see the classic work of Bunke [1] or the excellent work of Mohammed [2] for an introduction to consolidated strategies), much of the existing work is oriented towards recognition without the strict restraints of maximum duration (real time). In addition, such studies typically involve text on paper (or a similar material), which is significantly friendlier than the metal surface of a can. In conclusion, the priorities of an industrial character recognition system such as the one in question differ from common character recognition systems, and therefore, many of the usual solutions are not transplantable. An interesting historical overview of industrial OCRs can be found in Fujisawa [3]. A practical example of using new techniques in this context is found in Huangemail [4] and Nagarajan [5].

**Figure 1:**
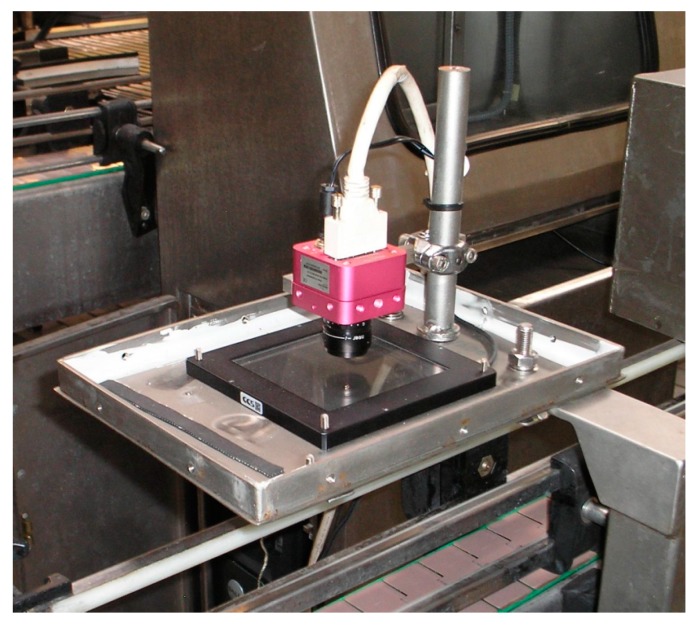
The plant system, which consists of a camera, illumination system, and support. The computer is in the background, and the conveyor belt system is underneath. The system is protected by a metal box not shown in this image that serves as a dark chamber.

**Figure 2:**
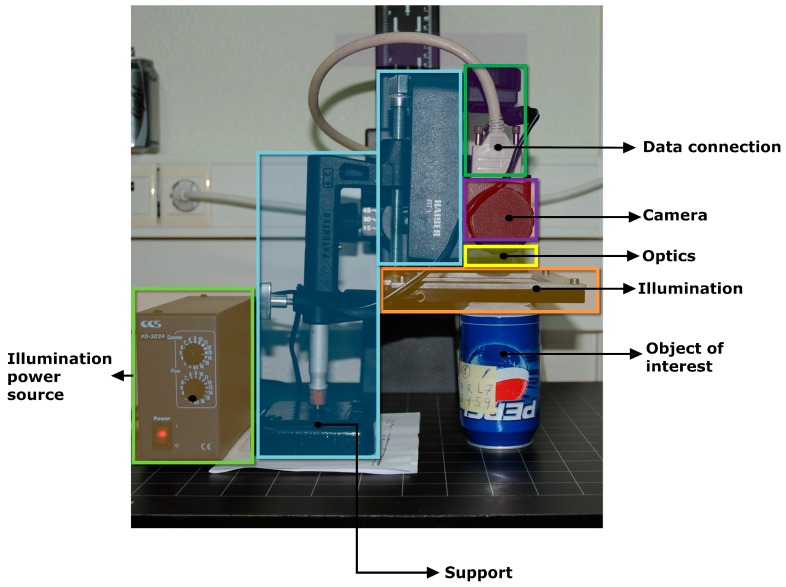
MONICOD components. Laboratory snapshot.

MONICOD is not a character recognition system because it does not identify text, which is advantageous. However, the operations it performs are demanding: validating 35 cans per second using an acquisition rate of 200 frames per second (an interesting case similar to MONICOD is outdoor license-plate recognition systems; in this scenario, the detection and identification of license plates has been achieved in less than 50 ms, *i.e.*, up to 20 frames per second can be processed; however, the results are highly dependent on the environment [6] with success rates of 90%–95% when conditions are favourable). MONICOD validates expiration codes on cans as presented elsewhere [7,8] and attempts to provide its own specialised solution (see Figure 1 and Figure 2).

During production, cans pass through the filling plant mounted on a conveyor belt moving at high speed. One of the production stages involves labelling the can with its expiration code. This code must be legible to the consumer. The printing system can fail for various reasons, which results in a defective (or blank) printing that can go unnoticed. MONICOD monitors the printing immediately after it occurs, and provides a warning when the printed code is unreadable. MONICOD consists of three stages:

1. The best frame is selected based on those available for a particular can.
2. Shapes are extracted from the selected frame and matched to the characters comprising the expiration date code.
3. The extracted expiration code is compared to the expected expiration code.

The traditional scheme, presented by Duda [9] and others, was adopted for this study. Several adaptations were implemented for validating the expiration code under the following conditions and restrictions:

(a) The validation of each can cannot be delayed more than a maximum time (1/35 of a second).
(b) The acquired images have low contrast (very dark) due to the short exposure time required for a 200 fps acquisition.
(c) The metallic surface of the can and the motion during image capturing result in unfavourable illumination (*i.e.*, non-homogeneous, glare, *etc.*).
(d) No shutter release (such as a photoelectric cell) is used to indicate when the can is centred in front of the camera.

This article describes the MONICOD algorithm chains for Steps 1 and 2. Its structure is as follows:

In Section 2—Detecting the optimum validatable frame—the most suitable frame for validation is selected out of several captured for any given can. Additionally, the area of the frame to be validated, *i.e.*, the area of interest, will be determined.

Next, in Section 3—Pre-proce ssing—the selected frame is pre-processed to obtain a swollen code and improve the overall image contrast.

Section 4—From pixels to fragments—describes how the ink is separated from the background to obtain the associated pixels known as ink fragments. These ink fragments might correspond to complete characters, parts thereof, or in the worst case, simply noise.

Section 5—Separation into lines: Assigning the fragments to lines of text—presents the method for determining to which line of code each fragment of ink corresponds. This method searches the interlinear spaces.

Section 6—Grouping fragments into shapes—details how the fragments of ink from Section 4 are turned into characters or discarded as noise. There are two criteria for this decision: only fragments belonging to the same line are associable (as determined in Section 5), and the “vertical overlap” between the fragments must be sufficient for association.

Finally, Section 7—Test—and Section 8—Conclusions—present some results and conclusions for the described techniques. 

## 2. Detecting the Optimum Validatable Frame

**Figure 3:**
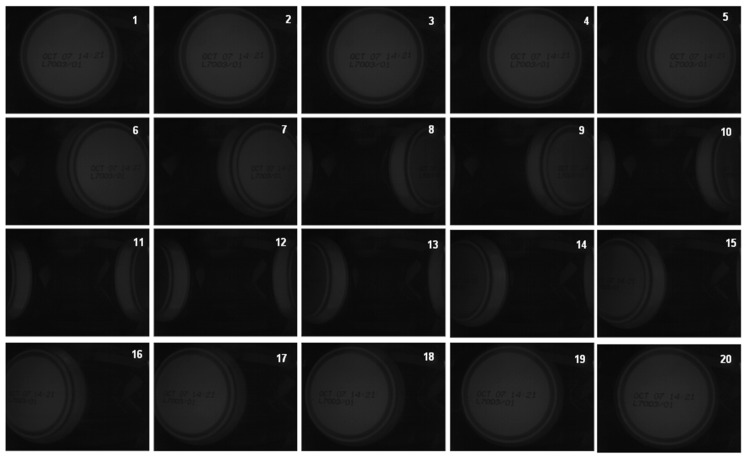
Acquisition during the transit of one can at 200 fps captures 20 frames. From left to right and top to bottom, a can in frames 1 to 10 leaves the field of view by the right side, while another can enters from the left in frames 10–20. Frames 5–15 are inadequate for validation.

During a continuous acquisition, not all of the acquired frames are useful for the validation (see Figure 3). It is necessary to distinguish the following:

(a) Which frames are “validatable”, that is, which frames contain a complete expiration code compatible with validation.
(b) Which of the “validatable set” of frames contain a can awaiting validation. If the frame contains an already validated can, it makes no sense to use it.
(c) Which frame in the “validatable set” that contains a can awaiting validation is optimal for validation. This step is important because each can will be validated using only one frame. The quality parameter for the chosen validation is the proximity of the expiration code to the centre of the frame.

### 2.1. Can Detection

MONICOD tracks each can’s approach based on one characteristic: the average illumination in a region. Promising tracking solutions with speeds of approximately 50 frames per second have recently emerged [10]; however, MONICOD operates at 200 frames per second with cans moving at great speed. Of note, we have two favourable conditions: cans are simple objects; and there are no occlusions, so the can is never out of sight.

Given a set of coordinates for the frame, which we call the area of interest, the activation percentage is defined as follows:

(1) $$P_{A}=\frac{N_{U}}{N_{T}}\times 100$$

where *N_U_* is the number of values in a threshold range [*U_A_*, *U_B_*], and *N_T_* is the total number of values in the area of interest.

**Figure 4:**
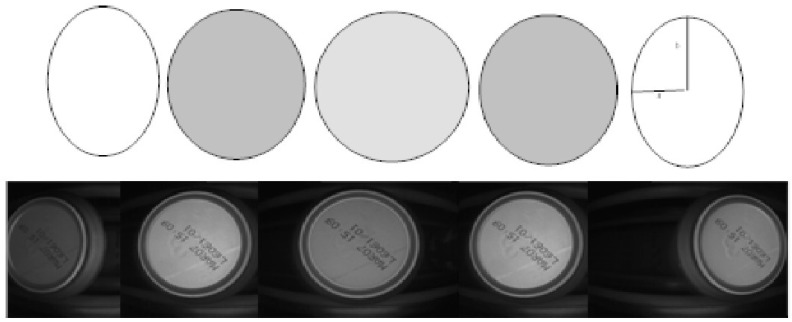
Elliptical areas of interest with different eccentricities according to their location. MONICOD uses the ellipse outline for detection and the area enclosed by the ellipse for extraction. This article describes a model using the circumference as the area of interest.

**Figure 5:**
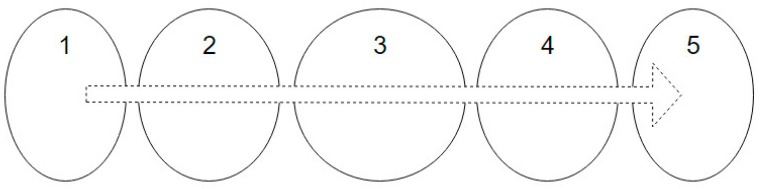
Natural order of activation with a forward direction from left to right.

For MONICOD, the area of interest for can detection is the circumference, or circular outline, with a configurable thickness and diameter slightly less than that of the can, as shown in the entry field. We worked with multiple, equidistant areas of interest that were located above the displacement axis of the cans within the entry field (see Figure 4 and Figure 5). The area which had the highest *P_A_* is called “dominant”. The centre of the dominant circumference was expected to estimate the centre of the can in the image.

### 2.2. States and Events

**Figure 6:**
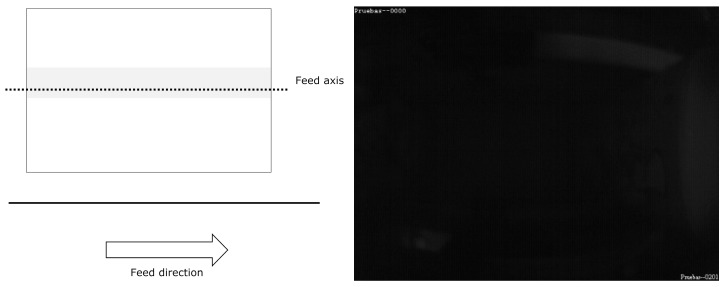
No event occurs. There are no cans in the entry field.

During can detection, we define two states; *No-can—*when no can is in the entry field as shown in Figure 6. The frame is assumed to be in this state immediately prior to the acquisition; and *Can*—when a can is detected in the entry field.

**Figure 7:**
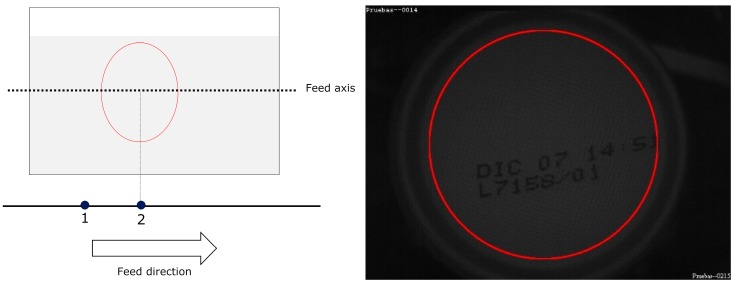
The *Can* state persists. There are no new events.

**Figure 8:**
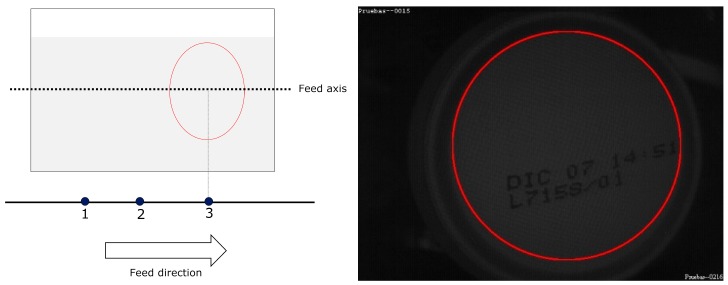
The *Can* state again persists. There are no new events.

**Figure 9:**
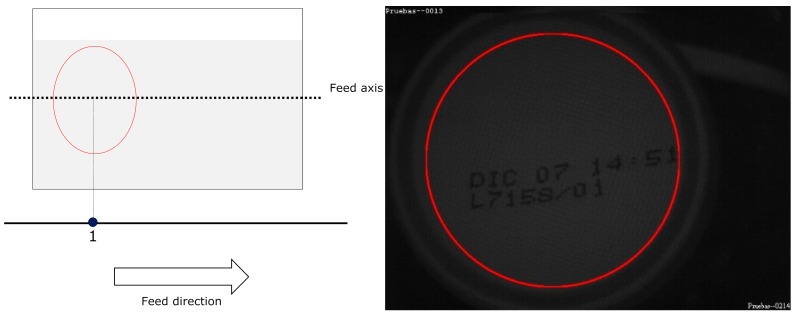
The “Enter” event occurs, and the system goes into the *Can* state.

**Figure 10:**
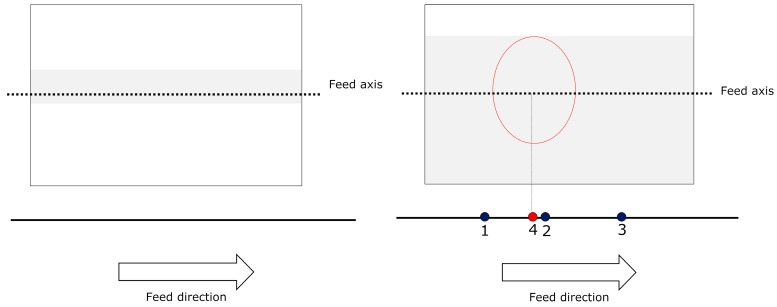
The figure on the left shows the Leave event, and the system enters the *no-can* state. The figure on the right shows a new *Enter* event with a fictitious *Leave* event, which closes the previous *Enter* event.

The transition between these two states is defined by the following events:

(a) There is no change of state. No event occurs (see Figure 6, Figure 7 and Figure 8).
(b) *Enter* Event. A new can enters the entry field. This is the transition from the *No-can* state to the *Can* state (see Figure 9). It is possible a previously detected can is still in the entry field.
(c) *Leave* Event. A can leaves the entry field. This is the transition from the *Can* state to the *No-Can*. A *Leave* state is also introduced when two consecutive *Enter* states occur and a can has not yet left the entry field before the other has entered. In this situation, a *Leave* event is lodged between two *Enter* events (refer to Figure 10).

In conclusion, the *Enter* and *Leave* events are paired and sequential. For every *Enter* event, a *Leave* event must follow.

### 2.3. Selection of the Can to Be Validated

Using these definitions, we distinguish the following:

(a) All frames between an *Enter* and *Leave* event are treatable. The rest of the frames are not treatable.
(b) All the frames between an *Enter* and *Leave* event belong to the same can. Each pair of *Enter-Leave* events increases the can counter.
(c) When a *Leave* event occurs, the dominant area of interest closest to the centre of the image is chosen as the optimal frame. This is justified because one expects the thin can will be the best centred in the entry field. The centre of the entry field has more homogeneous illumination and better optical conditions than the areas closer to the image borders.
(d) In addition, if an acquisition ends prior to an *Enter* event closed by a *Leave* event, the frames since the *Enter* event are discarded.

The optimal frame is sent to the validation system. In this way the validation system only works with the best frame available for a particular can (best centred in the entry field). No new acquired frames will be better for this particular can. The validation system never validates the same can more than once.

The validation system runs simultaneously to the procedure for detecting optimal frames. At 200 fps with 35 cans per second on the conveyor belt, the validation system must finish the validation of the can before receiving the next can for validation.

## 3. Pre-Processing

The fragmentation from ink jet printing and low image contrast mean the image being validated must be pre-processed. For this purpose, a simple enhancement operation is used: each pixel in the area of interest is replaced by the local minimum from its Moore neighbourhood (see Equation (2)):

(2) $$b_{i,j}=min\left(\begin{array}{ccc} a_{i-1,j-1} & a_{i-1,j} & a_{i-1,j+1}\\ a_{i,j-1} & a_{i,j} & a_{i,j+1}\\ a_{i+1,j-1} & a_{i+1,j} & a_{i+1,j+1} \end{array}\right)$$

**Figure 11:**
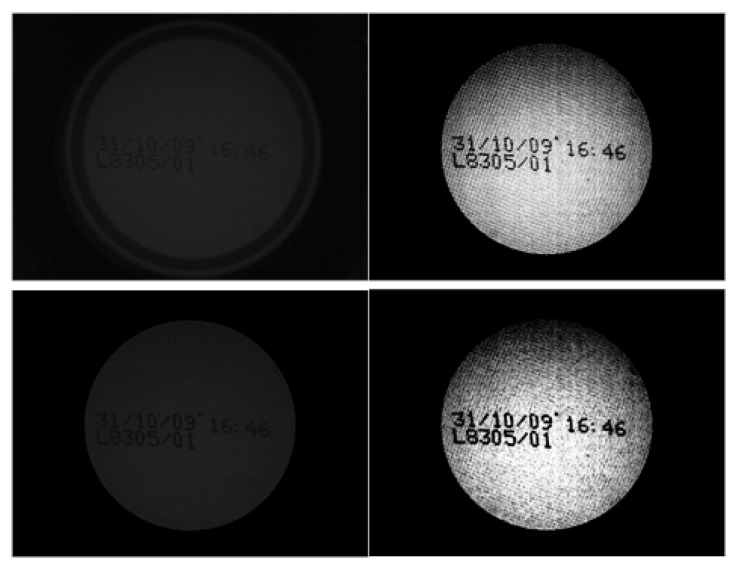
(**Top left**): original image. Top right: original image equalised; (**Bottom left**): original image enhanced; (**Bottom right**): original image enhanced and then equalised.

This operation has a low computational cost (it only involves comparisons) compared to other operations (such as convolution masks) that require additions and multiplications. In our experiments we found that applying a convolution mask severely limited our ability to fully validate a can before the next can to be validated arrived. Conversely, the operation that we chose to use did not compromise our goals and improved the image quite significantly, even to the naked eye (see Figure 11).

The time cost for our test machine of enhancement and equalisation, and the other stages of segmentation, is shown in Section 7. Next, an equalisation process is applied [11,12,13], and better use of the available grey levels counteracts the excessive darkness of the image. This is a popular, simple, and effective technique for revealing hidden details in low-contrast images. During implementation LUTs (look-up tables) are used to accelerate the equalisation process.

## 4. From Pixels to Fragments

Now we need to obtain fragments of character from pixels. First, the ink is separated from the background via a simple binarisation using a threshold. Currently, the threshold selection is performed during the calibration. In contrast to a manual threshold selection system based on visual inspection, automatic thresholding methods may be considered, such as those described by Otsu [14]. Sezgin’s study [15] is an interesting starting point that invites us to explore Kittler-Illingworth [16].

The pixels labelled as ink are formed into connected sets. Each set contains 0 (direct adjacency) or more ink pixels (indirect adjacency) connecting any two pixels in the set through a chain of direct adjacencies. Consequently, all of the connected pixels also belong to the set. From this description, every ink pixel is an element of one and only one set, which we call character fragments.

To turn pixels into fragments, we use an efficient implementation of a flood-fill algorithm: it begins with an ink pixel (the seed) and it calculates all the connected pixels that belong to the same set as the seed. The process repeats itself while there are pixels without ink not assigned to a set.

In this stage a fragment is a set of pixels that: (a) have been labelled as “ink pixels” and (b) are connected to each other. The characteristics of the fragment that are relevant for MONICOD are: the total number of ink pixels in the fragment, the *y* coordinate that is furthest to the EAST (*y*_max_), the *y* coordinate furthest to the WEST (*y*_min_), the *x* coordinate furthest to the NORTH (*x*_min_) and the *x* coordinate furthest to the SOUTH (*x*_max_). Because the flood-fill algorithm processes every point in each *fragment* *i* (*F_i_*), collecting the fragment characteristics during the image creation is very easy. These fragment characteristics include the number of pixels, centre, and the *x*_min_, *x*_max_, *y*_min_ and *y*_max_ coordinates. Many so-called “character fragments” correspond to “complete characters”, though not all. Therefore, a fragment-clustering stage is required. This procedure will be explained in the following sections.

## 5. Separation into Lines: Assigning Fragments to Lines of Text

In this stage, we attempt to label the previously collected fragments according to the code line to which they belong. It is important to know which line and position within a line a character belongs to because that determines its semantics. Grouping fragments into characters is also easier because only fragments from the same line are groupable.

In general, the difficulty of line detection is much less for printed text than for handwritten text because of its more regular shape and spacing between characters, words and lines. Lines of text are similarly oriented, and their tilt is also similar [17,18].

The line separation procedure searches the interline spaces because they serve as the line’s upper and lower boundaries. A line of text sits between either two interline spaces or the area of interest boundary and an interline space. If the expiration code consists of a single line of text, it is not necessary to separate the lines, and this phase is omitted.

### 5.1. Evident Interline Spaces

The previous step, pixels to fragments, identified ink pixels. It also identified which pixels belong to valid fragments and which do not. The amount of ink in each row is counted and increases for each valid pixel identified in a row. Rows with zero ink were identified as interline spaces. This identification was so rapid that we have called it “evident”.

### 5.2. Non-Evident Interline Spaces

**Figure 12:**
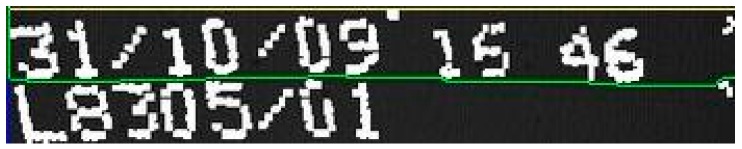
A divider is capable of separating two lines even though there are no rows with zero ink.

The previous approach is very simple and rapid. Unfortunately, noise or a tilted expiration code can obfuscate these evident interline spaces. In this scenario, an indeterminate number of independent agents are launched and compete concurrently to find dividing paths (see Figure 12).

**Figure 13:**
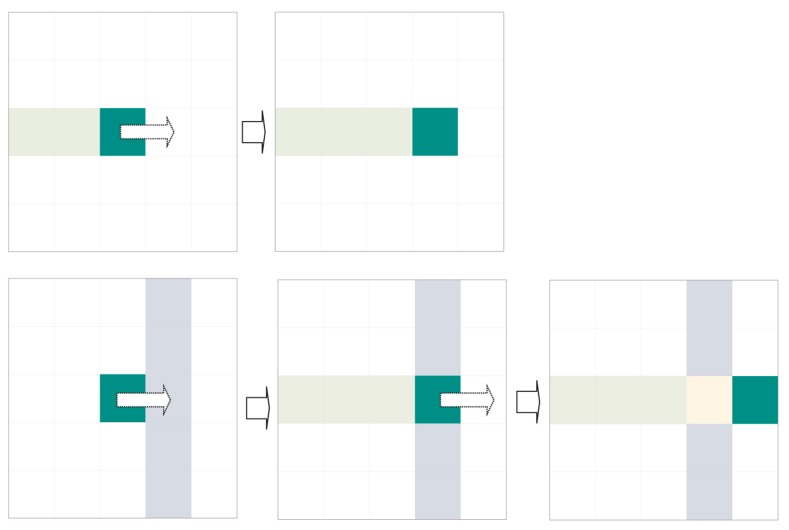
(**Top**) Forward movement straight in the direction of motion is favoured. The agent remembers the visited positions (the track); (**Bottom**) The agent ignores positions visited by agents travelling in the opposite direction.

We call these agents “dividers”. The dividers are identical to each other and follow the same displacement rules. Each divider has a preferred direction: either left to right (WEST TO EAST) or right to left (EAST TO WEST). Dividers differ from each other in their starting coordinates, which are unique for each one. Those with left-to-right preference start to the left (WEST) of the region of interest, while those with right-to-left preference start to the right (EAST) (see Figure 13).

Each divider keeps a log (or route log) detailing the coordinates it has travelled thus far. These coordinates cannot be explored by dividers with the same preferred direction. However, they are available for dividers with the opposite preferential direction.

Dividers only shift to legal contiguous positions in their preferred direction. A legal position is one (a) without ink; (b) that has not been accessed by another divider with the same preferred direction and (c) that is in the area of interest for extraction (see previous sections). If no legal positions are accessible by the divider, the divider performs mitosis.

**Figure 14:**
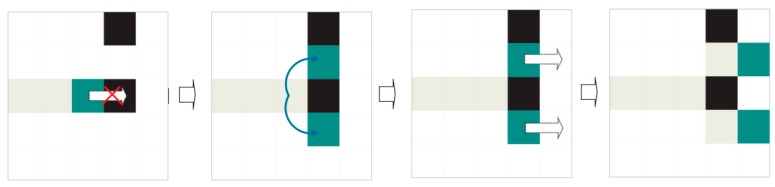
MITOSIS: Oblique locations are explored. Both are legal, and offspring are generated. The descendants inherit the position history of the progenitor and continue their progress. The progenitor simply disappears.

**Figure 15:**
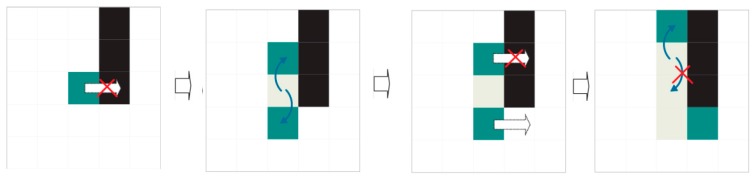
MITOSIS: The locations to the north and south are explored as a second step, thus generating offspring. In this case the southern agent advances but not the northern agent, which must perform a new mitosis.

**Figure 16:**
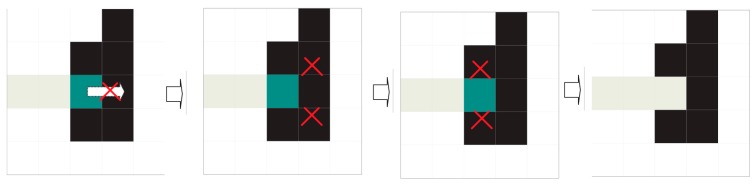
MITOSIS: An agent disappears (“dies”) without issue if it finds no alternative. The divider does not have offspring.

When mitosis, named for its similarity to natural cell division, occurs, the divider generates two copies with route histories identical to its own (see Figure 14 and Figure 15). Mitosis can fail (see Figure 16). The original divider then “dies”.

In its simplest form, the divider algorithm can be summarised as follows:

1. Advance straight in the preferred direction until reaching the other end or an illegal position that prevents progress and launches the division or mitosis procedure. A position is considered illegal if it contains ink or has already been visited by a divider with the same direction priority or the position is out of an area of interest for extraction.
2. The division procedure attempts to surround illegal positions to continue advancement. Division consists in creating new dividers with the same preferential direction as their progenitor and the same history of visited positions, to which the starting position will be added. A legal starting position is sufficient for a divider to be successfully created. Oblique mitosis is attempted first: attempting to create dividers in the northeast and southeast, if the preferential direction is WEST TO EAST, or in the northwest and southwest if the preferential direction is EAST TO WEST. If this fails (it has not been possible to create a divider), perpendicular mitosis is attempted: attempting to create dividers in the north and south. Finally, the divider dies whether mitosis has occurred or not.
3. The divider path length equals the detour cost produced by the division process. Thus, an unobstructed divider path has 0 cost (naturally such paths with 0 cost have already been filtered out by the previous search for evident interline spaces).
4. The first divider to reach its objective has the lowest-cost route and is the one chosen for the band division.

The complete method for two dividers would be similar to the following:

1. Select the border or fringe where the procedure will be applied.
2. Select a promising starting row.
3. Two dividers are launched simultaneously, one with left-to-right priority and the other with right-to-left priority, using the same starting row (Step 2) as the point of origin; however, each starts on opposite ends. Their histories will be initialised with a blank route.
4. A single exploration step is made if the dividers remain alive:
  (a) The existing dividers execute the divider procedure once.
  (b) If a divider reaches the end opposite its starting point, the exploration is declared successful.
5. If the exploration procedure ends with success, the divider route is the band border, and the procedure is abandoned.
6. If the exploration procedure fails, the next optimal starting row is selected, and we return to step 2. After *n* failures, the interline operation has failed, and validation is NOT possible.
7. We return to Step 4.

In the current implementation, the procedure for selecting a promising starting row selects the row with the minimum amount of ink that is at an acceptable distance from the limits established in Step 1. The acceptable distance used is the minimum height of a character.

## 6. Grouping Fragments into Shapes

In general, the most common inter-character space of a fragmented character is horizontal. Assuming the code is perfectly aligned with the reading axis, there will be many opportunities to regroup such characters based on the overlap of their fragments on the *y*-axis.

### 6.1. Overlap of Projections on the y-Axis

Two shapes on the plane, *A* and *B*, have *y* values to their right and left of $y_{\max}^{A}\;y_{\max}^{B}\;y_{\min}^{A}\;y_{\min}^{B}$. As a result, $y_{\min}^{A}\leq y_{\max}^{A}$ , and $y_{\min}^{B}\leq y_{\max}^{B}$. We say *B* overlaps with *A* if at least one of the following conditions is fulfilled:

(3) $$y_{\max}^{A}\leq y_{\max}^{B}\leq y_{\min}^{A}$$

(4) $$y_{\max}^{A}\leq y_{\min}^{B}\leq y_{\min}^{A}$$

The direction of overlap (*A* overlaps with *B* or *B* overlaps with *A*) is irrelevant to this method. We can associate the overlap magnitude to the grouping strength.

### 6.2. Fusion

Two conditions must be satisfied for fusion:

1. The fusion must be designated as “legal”. That is, the involved fragments are part of the same band, and the fragment fusion does not exceed the maximum width or amount of ink possible for a character.
2. There is sufficient vertical overlap between the fragments (or groups of fragments).

Fragments comprising a character do not always display vertical overlap, and such cases are invisible to this method. The pre-processing enhancement moderates such situations. Furthermore, groupings based on vertical overlaps stop being feasible when the code is tilted over 10°.

### 6.3. Fission

A printing with an unusually thick trace combined with the described enhancement procedure can suppress the inter-character spaces and cause different characters (or parts thereof) to be joined into a compact mass of ink. These masses accrue into “super-fragments” during the described ink association process, which leads to two problematic circumstances: the ink forms a bridge between characters, or there is no real ink between the characters but their thickness (pre-processing + flood-fill threshold) causes them to merge. The latter problem can be addressed by preserving the post-process entry field, where the separation still existed. Using that background information, we can reassign the set points to two different sets and thus overcome this anomaly (fission).

The first step is detecting these super-fragments. There are two indicators: excessively broad (wide) fragments and excessive ink quantities in a fragment.

A width greater than the widest expected printed character and/or an amount of ink greater than the printed character with the greatest expected amount of ink indicate that this is a super-fragment. These values are obtained by calculating *y*_min_ and *y*_max_ and the number of ink pixels on the characters mentioned during calibration time. Once the situation has been detected, the following procedure occurs:

1. Given the original image and LUT equalisation table (grey substitution table) already generated (it was not destroyed), we equalise the super-fragment coordinates. There is NO enhancement.
2. We only associate ink in the super-fragment coordinates.
3. It is NOT necessary to assign any bands. All of the fragments inherit the band assigned to the super-fragment.
4. The original super-fragment has disappeared, and we must have common fragments in its place. If the super-fragment persists, the characters must be joined at the source, which is a legitimate defective printing.

### 6.4. The Grouping Cycle

**Figure 17:**
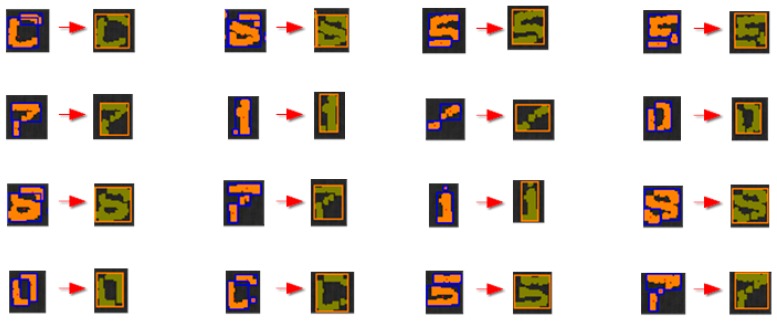
Grouping examples from the MONICOD experiments.

The grouping cycle is an iterative process that groups fragments with an ordered shape. It is the heart of fragment grouping (see Figure 17 for the results).

The cycle crosses all non-grouped fragments (and any already grouped fragments), calculating the superpositions of “legal” projections on the *y*-axis. “Illegal” groupings are assigned a null overlap.

The information obtained from grouping attempts are performed on the non-grouped fragments (and already grouped fragments) following a strict order based on their overlap magnitudes. A previously grouped fragment in the current cycle cannot be re-considered for grouping within the same cycle. If no more groupings are possible, the overlap table is recalculated, and the grouping process is repeated. If all grouping attempts in a cycle fail, the iterative process is terminated.

**Figure 18:**
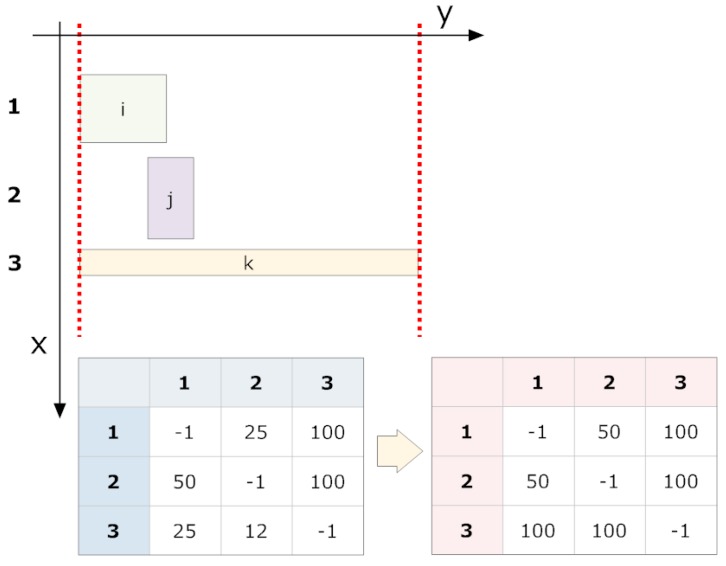
Schematic of a grouping. We proceed based on three groups: (1)–(3). Each group contains a single fragment. The first cycle of grouping starts on the left. Based on the overlap table, a simplified version is generated where only the maximum magnitudes are listed (this table is symmetric). In this example, the scan order is from top to bottom and left to right (it could be any order) so that (1) and (3) are first eligible for grouping. Therefore, (2) may not be grouped with either (1) or (3) because they have been grouped in this cycle.

**Figure 19:**
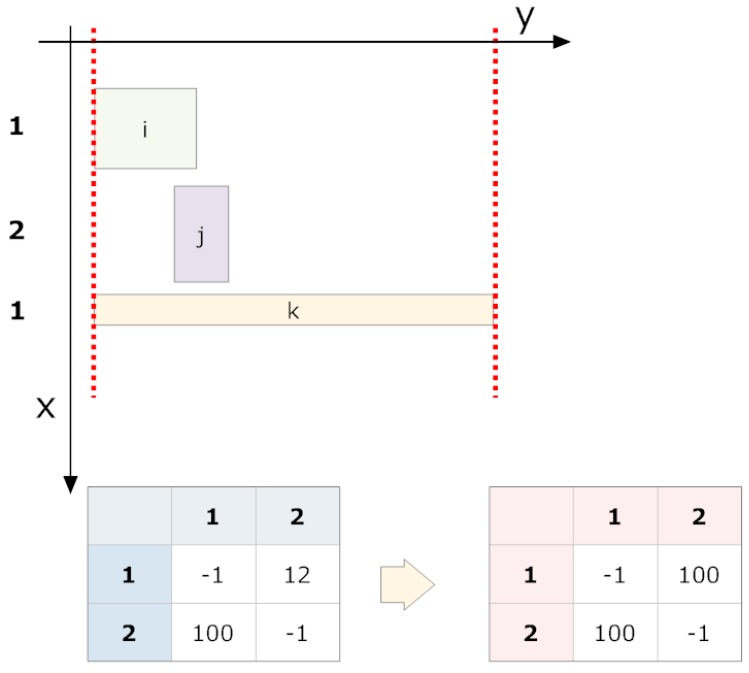
The second grouping cycle located on the right consists of group (1), which now contains two fragments. The described procedure is repeated.

1. The table of overlaps is initialised with each row and column corresponding to a fragment (now a group) within the same band.
2. The overlap table is calculated (see Figure 18 and Figure 19).
3. The pair (*i*, *j*) with the maximum overlap is chosen from the table, where *i* and *j* have not been successfully grouped in the current cycle. If none exists, we go to step 5.
4. A grouping attempt is made with the pair (*i*, *j*).
  (a) If pairing (*i*, *j*) is successful, it now forms a fragment group and will no longer be considered individually. Neither *i* nor *j* are eligible for new groupings in this cycle. We return to Step 3.
  (b) If the grouping fails, we return to Step 3.
5. If at least one grouping attempt succeeded, we return to Step 2. If not, the grouping cycle is terminated.

The grouping system successfully dealt with most common cases, especially when the code had no slope (see Figure 17). Examining the successful grouping samples shows almost all of the cases consisted of two clusters, with a few exceptions having three. A vertical overlap was almost always observed and ultimately was key to the grouping mechanism. Some characters are more likely than others to fragment, and their fragmentation points are almost always the same. A particularly problematic case is the forward slash (/). This fragment has no vertical overlap, and its slenderness leads to fragmentation with over two fragments.

### 6.5. The Acquired Character

This process yields a list of one or more fragments for each character. The following parameters are attached: centre, number of points, the band to which it belongs, and both the maximum and minimum *x* and *y* coordinates. In this way, an elaborate vector of characteristics is obtained that is robust to variances such as those obtained using calculus of moments [19]; however, MONICOD directly treats the shape.

### 6.6. Ordering of Fragments by Order of Reading

The interpretation of each character in the code depends on its position in the code. By convention, MONICOD orders the acquired characters for reading from left to right, top to bottom. This is conducted in two phases.

1. The characters are grouped by their bands, which are ordered from top to bottom.
2. The characters in each band are ordered starting with the minimum y coordinate.

### 6.7. The Output

**Figure 20:**
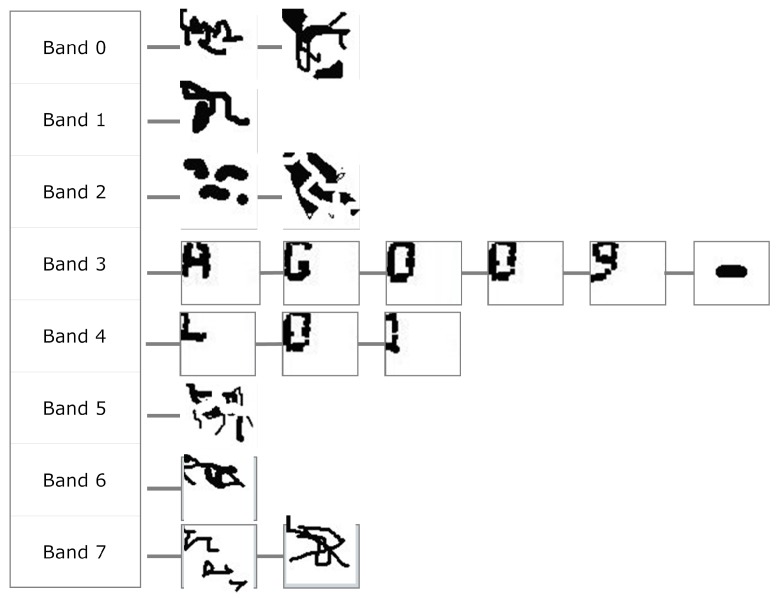
Structure retrieved from the can by the shape extractor.

The actual code is the acquired character structure as shown in Figure 20. We still do not know if the extracted shapes are actually code characters, or even characters of any language. They are the segmentation results, which are expected to contain some amount of printing or acquisition noise.

The final stage of MONICOD, to explain it briefly, applies the available knowledge of the expected code to the structure of Figure 20. It is known for example that the first line of the expected code has five characters, so we can rule out bands 0, 1 and 2 containing, respectively, 2, 3 and 2 extracted shapes.

Band 3 already has enough extracted shapes to start comparing them with the expected shapes: the “A” character in this case. The comparison will be positive with the first shape extracted from band 3. Then it compares the second expected character with the next extracted shape to be compared; *i.e.*, the second shape extracted from the extracted band (“G”). In this case it is successful. It will then search for the next expected character (“O”) in the following extracted shape, and so on. If it is determined during the process that the expected shape is not in the expected place, the process stops and Negative Validation is declared.

Note that MONICOD does not attempt to recognise the extracted shapes. Instead it attempts to validate the extracted shapes against the expected shapes following the order of reading. With the described procedure MONICOD tolerates the existence of extracted shapes (noise) that are not expected characters as long as all the expected shapes are in the expected order. This is feasible because it has been observed empirically that (a) the noise obtained this way never looks like legal characters and (b) the noise does not compromise the readability of the code. This has been observed to be so by us in our research. This process, along with the algorithm for comparing expected and extracted shapes, will be described in detail in a future article.

## 7. Test

An Intel(R) Xeon(TM) CPU 3.80 GHz 2.00 GB of RAM (dual-core) machine running Windows XP SP3 32-bit was used for these tests. The sample we worked with was extracted directly from a canning plant and stored on a disc to allow the same experiments to be repeated as often as necessary. It was manually verified that the cans in the sample are all CORRECTLY labelled.

The stored “video” (9885 frames) corresponds to five independent 10-s acquisitions. Larger acquisitions were not possible for technical reasons regarding storing the acquisition while it occurs. Each acquisition was made at 200 frames per second (2000 frames per extraction). The acquisition time was approximately 20 min.

An uncompressed image format was used for the data storage to avoid introducing artefacts, colour degradation or frame loss due to the compression calculation (900 kb per frame). The image resolution was 640 × 480 with 8-bit colour depth (grayscale). The camera operated at 200 frames per second with an exposure time of 0.990 ms. The sample occupied 8.48 GB of disc space.

The sample was divided into two sets:

- The learning set was composed of 1005 frames, *i.e.*, approximately 5 s of non-continuous acquisition. This set was constructed by merging excerpts from each of the five acquisitions (201 frames for each acquisition). These frames maintain the chronological order of the acquisition. It was manually verified that the set contained 53 cans. At 200 frames per second, the acquisition was 5.025 s in duration.
- The validation set was composed of 8884 frames, *i.e.*, approximately 45 s of non-continuous acquisition. This set maintains the chronological order of acquisition. It was manually verified that the set contained 465 cans. At 200 frames per second, the acquisition was 44.42 s in duration.

The separation between learning set and validation set was used in the final stage of MONICOD. In this final stage MONICOD uses the learning set to learn what shapes are expected during the learning phase. In this learning phase the system is limited to labelling extracted shapes with supervised expected codes and stores these segmented shapes in its memory as “standard” templates. In this phase there is NO validation. It is important that the learning set is truly representative. In normal operating mode it compares these learned shapes with those obtained from the validation set. The comparison process is based on two tests: (a) a comparison of invariant characteristics (such as total amount of ink) and, if the first test establishes sufficient similarity; (b) the extracted standard template is compared with the stored standard template. If the second test is passed, the extracted shape is validated positively. This process is repeated methodically for each of the characters of the code. This way the expiration codes are validated. This process could be improved by allowing the system to also learn in normal operating mode. In any case, a detailed description of this final stage is beyond the scope of this article.

The expiration date format used for developing and testing MONICOD is divided into two lines of text. Each line of text may contain between four and ten characters. Characters may be upper case letters, numbers and the characters “/” and “:”. It is an actual format used by Compañía Cervecera de Canarias and Compañía Embotelladora de Canarias (see Acknowledgments).

**Figure 21:**
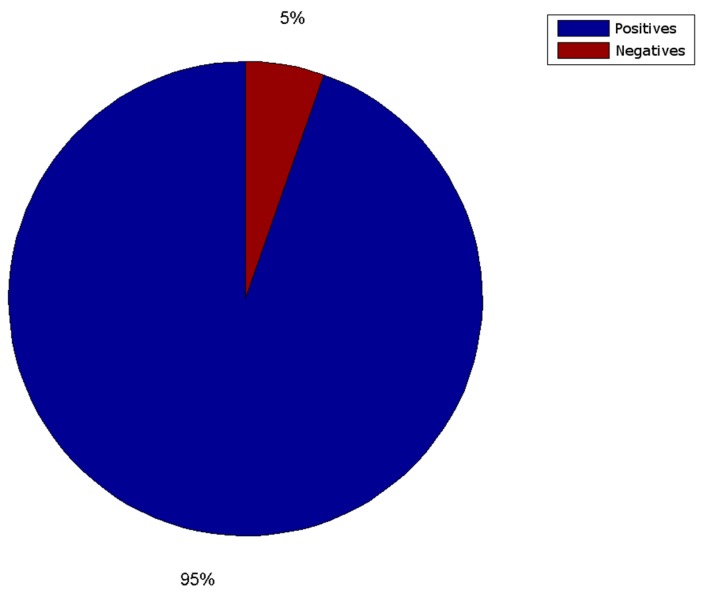
Successful validations are blue (95%). False negative validations are red (5%).

**Figure 22:**
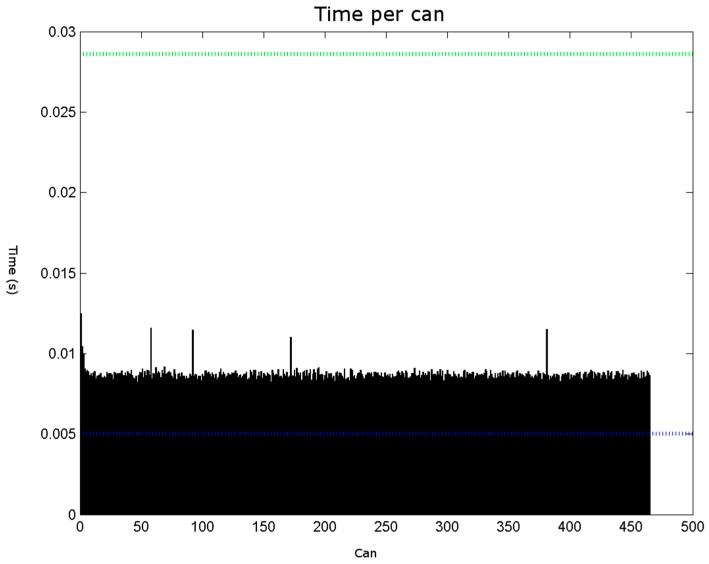
The time spent on each sample can is shown in seconds. The blue line corresponds to 1/200 s. The green line corresponds to 1/35 s. The shape extraction is fast enough to process 35 cans per second but insufficient to address all of the acquired frames.

**Figure 23:**
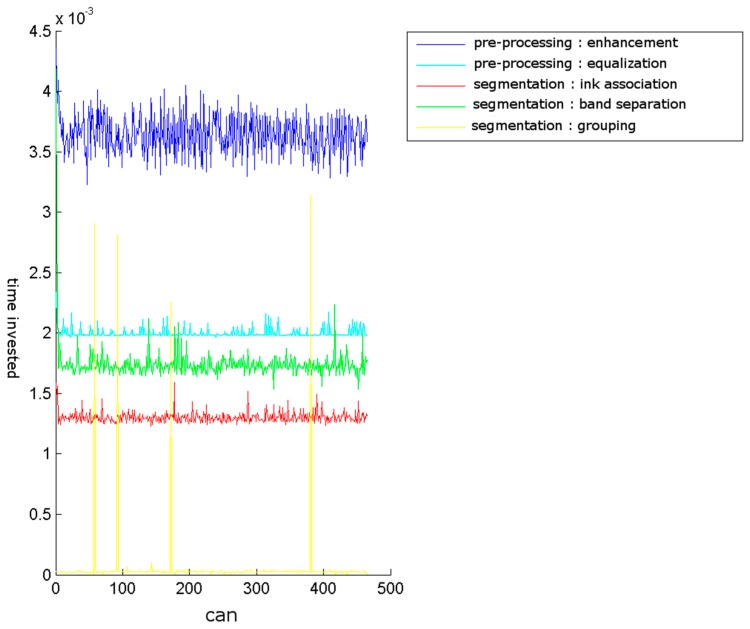
Breakdown of the times by stages. Enhancement and equalisation, the two pre-processing phases, were the most time-consuming. The grouping stage had considerable ups and downs because it depends on the number of clusters required. Each grouping represents a new grouping cycle. Too many groupings indicate an incorrect system calibration.

**Figure 24:**
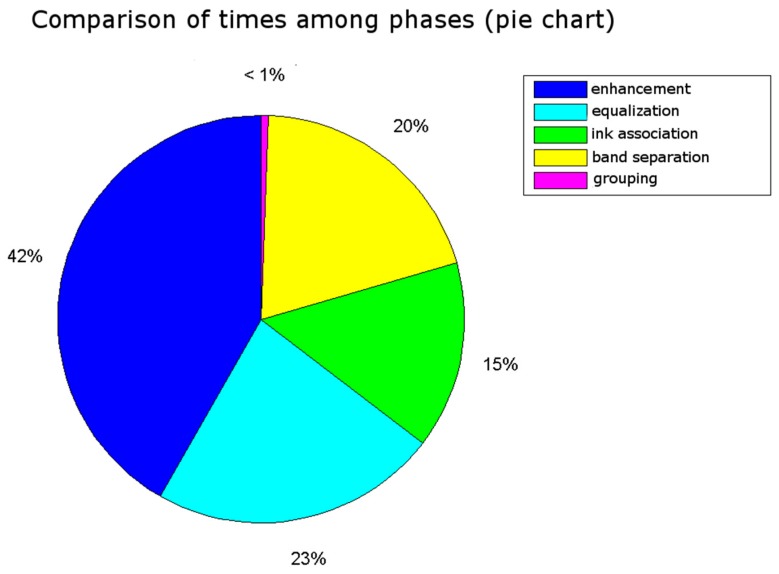
The time taken by each phase indicates enhancement is the most time-consuming phase followed by equalisation. Both phases correspond to the pre-processing step.

The obtained results are shown in Figure 21, Figure 22, Figure 23 and Figure 24. The conducted tests found a number of false negative validations (5%), which is a high error rate. However, they also show that the system is nearly three times faster than required. No specialised hardware was used. We believe that this advantage should provide room for quality improvements.

Table 1 was obtained to examine why the false negative validations occurred.

The techniques described in Section 2, Section 6 and Section 7 do not guarantee a 100% success rate. The optimum validatable frame detection algorithm (Section 2) was applied to frames that had not been pre-processed (Section 3). It is very sensitive to lighting conditions and any possible slack on the conveyor belt. A can may be shunted slightly so that is too high or low in the frame. In these cases the code characters may end up outside of the area of interest for extraction.

The line separation algorithm (Section 5) will only function correctly if the code on the can is not too tilted (less than 10%). Unfortunately cans may be jolted while on the conveyor belt, which causes them to turn slightly. If this occurs after printing and before acquisition we may find codes that are too slanted. The best solution is simply to physically move the printing and acquisition points closer together or to locate them at the same place. This was the solution adopted by us and it allowed us to maintain the slant below the indicated threshold.

Grouping errors (Section 6) were rather more marginal. The main reason for this was the “unfortunate” misprints that, although they did not compromise the readability of the code, produced undesirable groupings or cancelled out legal groupings. These errors prevented the correct formation of some characters from their fragments.

Any of these failures lead to false negative validations. This means that MONICOD sets off an incorrectly printed code alarm, when in fact it is correctly printed. We consider that solving or alleviating these problems should be the focus of future work.

## 8. Conclusions

MONICOD validates expiration codes on beverage cans and is capable of operating at high speeds. This article presents two portions of this process: the frame selector that validates each can and the shape extractor that obtains the text to be validated.

This system operates using a general-purpose machine without requiring special hardware. There are no specific software requirements, and various systems have been tested (Windows, GNU/Linux, MAC OSX). One dependency does exist, *Sapera LT*, which is the suite of libraries from *Teledyne Dalsa* that MONICOD uses to establish a link to the acquisition card; these libraries only operate on Windows, making it the default operating system.

MONICOD combines different paradigms and strategies: simplicity, integer arithmetic using simple operations (sums and products), multi-threaded architecture, a favourable physical configuration (which eliminates the possibility of capturing rotated cans images, for example), the use of escape conditions (e.g., not finding sufficient ink or not demarcating the necessary lines of code, which is interpreted as a printing error and prevents the rest of the validation steps from proceeding).

MONICOD easily meets the time demands; however, its validation quality (with 5% false negative validations) compels us to consider improvements. The main causes of these failures were identified, and the following improvements are being considered:

(1) Refining the optimum validatable frame detection.
(2) Applying a thresholding by zones rather than globally as is currently done, which reduces the enhancement intensity. See Zhu [20] for an example. The enhancement thickens the code and can obscure the interline spacing.
(3) Moving the camera closer to the printing system to dampen the effects of code tilting caused by the can turning.

Given the extreme nature of the starting specifications (an unfavourable illumination combined with high speed) and the achieved hardware/software independence, we believe MONICOD can provide a family of industrial validators capable of dealing with expiration codes in formats other than cans.

As we have already mentioned, our study is intended to be applied to the monitoring of labels on a production line in the beverage can packaging industry. There is no intrinsic limitation on applying the described methods to other contexts. The only possible exception to this is the optimum validatable frame selector. This selector uses the circular shape of the base of the can to determine the area of interest. However, this selector could be easily adapted to a much wider range of shapes, depending on the case in question. The area of interest could, for example, be square, rectangular, an irregular polygon, *etc*. Consequently, the authors consider that the system described in our article can be applied to any scenario requiring recognition (or validation) of printed characters, industrial or otherwise. It is quite possible, however, that key modifications would have to be made depending on the chosen font or spelling system, or whether the text layout is horizontal or not (e.g., vertical Asian scripts).

In addition, the algorithms are sufficiently independent of each other to be used separately, in combination with other techniques: for example, the line separation algorithm or the character fragment grouping system. Other researchers may benefit from this paper by incorporating one or more of the algorithms into their own character segmentation applications.

It is expected that in other contexts with less unfriendly surfaces (e.g., cardboard or paper) that are not affected by the reflected glare from metal surfaces, the results would be even better. However, it should be noted that the algorithms described in this paper are designed for applications where the speed of segmentation takes priority over the quality. If quality was more important and speed not a critical factor, then other methods may be more appropriate.

Regarding the comparison with other methods, we would like to point out that the purpose of MONICOD is to operate relatively smoothly with printed text at high speed, in adverse conditions with additional character noise. Traditionally, studies on character recognition have emphasised recognition quality: handwritten text and Asian scripts, for example. Accordingly, the criteria used to distinguish the quality of work relate more specifically to recognition success rates than the number of characters recognised per second. Moreover, the characters validated by MONICOD are printed on wet metal surfaces (metallic glare). This is an unusual surface for character recognition problem-solving. There are some specialised commercial solutions available, but they are closed-loop solutions and there is very little available information that would allow us to make the appropriate comparisons. Some of these solutions are limited to verifying whether there is a code on the can or not. For this type of verification true character segmentation is not required.

## Figures and Tables

**Table 1:** Breakdown of the false negative validations according to their cause.

Cause of the Failure	% of Total Failures	Description
Failure to detect the optimum validatable frame.	52%	The complete code was not within the area of interest.
Failure to separate lines.	44%	The code was not correctly separated into text bands/lines.
Failure to group the fragments into shapes.	4%	The grouping was incorrect.
